# Fenofibrate Interferes with the Diapedesis of Lung Adenocarcinoma Cells through the Interference with Cx43/EGF-Dependent Intercellular Signaling

**DOI:** 10.3390/cancers10100363

**Published:** 2018-09-28

**Authors:** Katarzyna Piwowarczyk, Edyta Kwiecień, Justyna Sośniak, Eliza Zimoląg, Emiliana Guzik, Jolanta Sroka, Zbigniew Madeja, Jarosław Czyż

**Affiliations:** Department of Cell Biology, Faculty of Biochemistry, Biophysics and Biotechnology, Jagiellonian University, Gronostajowa 7, 30-387 Kraków, Poland; edytaaa.kwiecien@gmail.com (E.K.); sosniakjustyna@gmail.com (J.S.); eliza.zimolag@uj.edu.pl (E.Z.); emiliana.guzik@gmail.com (E.G.); jolanta.sroka@uj.edu.pl (J.S.) z.madeja@uj.edu.pl (Z.M.)

**Keywords:** fenofibrate, lung cancer, diapedesis, Cx43, EGF

## Abstract

Extravasation of circulating cancer cells is regulated by the intercellular/intracellular signaling pathways that locally impair the endothelial barrier function. Co-cultures of human umbilical vein endothelial cells (HUVECs) with lung adenocarcinoma A549 cells enabled us to identify these pathways and to quantify the effect of fenofibrate (FF) on their activity. A549 cells induced the disruption and local activation of endothelial continuum. These events were accompanied by epidermal growth factor (EGF) up-regulation in endothelial cells. Impaired A549 diapedesis and HUVEC activation were seen upon the chemical inhibition of connexin(Cx)43 functions, EGF/ERK1/2-dependent signaling, and RhoA/Rac1 activity. A total of 25 μM FF exerted corresponding effects on Cx43-mediated gap junctional coupling, EGF production, and ERK1/2 activation in HUVEC/A549 co-cultures. It also directly augmented endothelial barrier function via the interference with focal adhesion kinase (FAK)/RhoA/Rac1-regulated endothelial cell adhesion/contractility/motility and prompted the selective transmigration of epithelioid A549 cells. N-acetyl-L-cysteine abrogated FF effects on HUVEC activation, suggesting the involvement of PPARα-independent mechanism(s) in its action. Our data identify a novel Cx43/EGF/ERK1/2/FAK/RhoA/Rac1-dependent signaling axis, which determines the efficiency of lung cancer cell diapedesis. FF interferes with its activity and reduces the susceptibility of endothelial cells to A549 stimuli. These findings provide the rationale for the implementation of FF in the therapy of malignant lung cancers.

## 1. Introduction

Lung cancer is one of the most frequently diagnosed cancers in Europe [1,2]. The five-year survival rates of the patients with non-small-cell lung cancers (NSCLCs) are relatively low and reach 24% and 4% for advanced and metastatic NSCLCs, respectively. The high lethality of NSCLCs predominantly results from the local and systemic dissemination of cancer cells through the respiratory and vascular system. The metastatic cascade of NSCLC leads to the progressive deterioration of respiratory functions and results in the formation of secondary tumors in the brain, kidneys, and bones. NSCLC malignancy is determined by intrinsic invasive properties of cancer cells and extrinsic signals from the local tumor microenvironment. Cancer cells acquire invasive properties in the process of epithelial-mesenchymal transition (EMT) [3]. It elevates their capability of penetrating tissue barriers and of entering the circulation in a process of diapedesis [4,5,6]. Notably, the interrelations between the invasiveness and drug-resistance of cancer cells have been reported. They are responsible for the relative inefficiency of currently available therapeutic approaches against malignant lung cancers. Consequently, drug-resistant cancer cells selectively disseminate throughout the organism, leading to cancer relapses after the cessation of treatment. These facts stress the necessity for the elaboration of new anti-cancer strategies that would bypass the limitations of conventional chemotherapy. Because the speed of diapedesis limits the efficiency of the malignant dissemination of cancer cells [7,8], cancer cell diapedesis is a promising therapeutic target.

During the diapedesis, circulating cancer cells progressively adhere to the endothelium and locally activate endothelial cells via paracrine and juxtacrine signaling [8]. The efficiency of endothelial activation is determined by the quality and quantity of intercellular communication networks constituted between cancer and endothelial cells [8,9,10]. Paracrine, juxtacrine, and gap junction-mediated signals disrupt the endothelial layer in the proximity of cancer cells, thus facilitating their selective transmigration and homing in adjacent tissues. The active contribution of endothelium in cancer cell diapedesis [9,11] opens perspectives for introducing the drugs that would interfere with the intercellular signaling involved in endothelial activation. Prospectively, such a strategy would help to bypass the drug-resistance of cancer cells.

Fenofibrate (propan-2-yl 2-{4-[(4-chlorophenyl) carbonyl]phenoxy}-2-methylpropanoate; FF) has recently been suggested for metronomic strategies targeted at the diapedesis of circulating cancer cells [12,13,14,15,16,17,18]. FF is an FDA-approved vasoactive drug [19,20], applied to normalize serum levels of triglycerides and cholesterol, and to improve the HDL:LDL ratio [21,22,23,24]. In addition to the “canonical” (hypolipidemic) activity of FF, its interference with neoplastic and invasive properties of cancer cells has been described. We have previously demonstrated that FF attenuates endothelial susceptibility to the signals generated by prostate cancer cells. Thus, FF reduces the efficiency of prostate cancer cells diapedesis [9]. FF also interferes with primary tumor vascularization and the subsequent metastatic cascade via cytostatic effects on endothelial cells [19,25,26,27,28,29]. Multifaceted FF activities are related to the activation of peroxisome-proliferator activated receptor-α (PPARα) and to the PPARα-independent elevation of reactive oxygen species in the cells (ROS; [30,31]). They prompted us to test the suitability of FF for the metronomic chemotherapy of advanced lung cancers.

Co-cultures of human umbilical vein endothelial cells (HUVEC) with cancer cells enable the recapitulation of the events associated with the diapedesis of lung cancer cells. We used this experimental approach to estimate the effect of FF on the efficiency of this process. In particular, we analyzed (i) the role of paracrine and Cx43-mediated gap junctional intercellular communication (GJIC) [32] between HUVECs and lung adenocarcinoma A549 cells during their diapedesis. We also (ii) identified the mechanisms underlying the interference of fenofibrate with these systems. Finally, we (iii) estimated the consequences of FF for the selectivity of endothelial barriers during lung cancer cell diapedesis.

## 2. Results

### 2.1. FF Interferes with the Diapedesis of A549 Cells in a PPARα-Independent Manner

**Figure 1:**
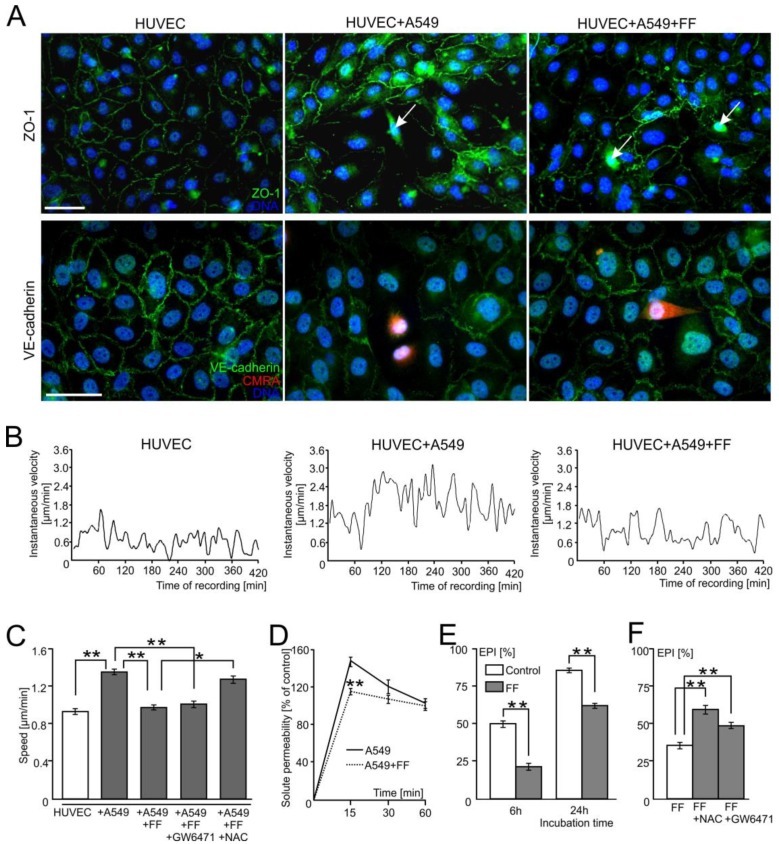
Fenofibrate inhibits the penetration of the endothelial layer by lung adenocarcinoma (A549) cells. Human lung cancer A549 cells (stained with CMRA-Cell Tracker or marked with arrows) were seeded onto human umbilical vein endothelial cell (HUVEC) monolayers at the density of 1300 cells/cm^2^. The integrity of endothelial continua, HUVEC motility, and endothelial permeability was assessed by anti-ZO-1/VE-cadherin immunostaining ((**A**); 6 h), time-lapse videomicroscopy ((**B**,**C**); 7 h), and solute permeability tests (**D**), respectively, in the absence or in the presence of 25 μM fenofibrate (FF). (**B**,**C**) shows instantaneous speeds of single HUVEC in control conditions and in the presence of A549/25 μM FF, and the comparison of average speeds of HUVEC movement in the presence of A549 cells/FF/GW6471/NAC, respectively, quantified based on changes in cell centroid position. (**E**) The effect of FF on the efficiency of A549 diapedesis (TransEndothelial Penetration; EPI) was estimated after 6 and 24 h of co-culture. (**F**) Effect of PPARα inhibitor (GW6471) and ROS scavenger (N-acetyl-L-cysteine; NAC) on A549 diapedesis (EPI), estimated after 6 h of A549/HUVEC co-culture. Transmigration of at least 200 A549 cells was analyzed for each group. The statistical significance of the differences in (**C**–**F**) was tested with one-way ANOVA followed by a post-hoc Tukey’s HSD comparison (* *p* < 0.05 and ** *p* < 0.01). Error bars represent SEM. All results are representative of at least three independent experiments (*n* ≥ 3). Scale bar = 40 μm. Note that the relatively efficient diapedesis of A549 cells is considerably inhibited by FF.

Human lung adenocarcinoma A549 cells induce the local disruption of endothelial integrity. This is illustrated by the disruption of tight (ZO-1^+^) and adherens junctions in the regions of endothelial layers situated close to CMRA-labeled cancer cells (VE-cadherin^+^; Figure 1A; Video S1). In addition to HUVEC retraction, time-lapse videomicroscopy studies demonstrated the activation of endothelial cells in the proximity of A549 cells. This is illustrated by increased instantaneous speeds of single HUVECs in contact with A549 cells (Figure 1B) and increased averaged values of HUVEC speed of movement, calculated at the population level (Figure 1C). A total of 25 μM fenofibrate (FF) exerted an inhibitory effect on A549-induced HUVEC motility (Figure 1B,C and Figure S1A) in the absence of any discernible effect on the motility of A549 cells [33]. Concomitantly, FF counteracted a local disruption of endothelial continuum in the proximity of A549 cells, as revealed by immunofluorescence (Figure 1A), time-lapse videomicroscopy (Video S2), and solute permeability tests (Figure 1D). These vasoprotective effects of FF were correlated with the inhibition of A549 diapedesis observed in the presence of FF. Averaged values of the transEndothelial Penetration Index (EPI) decreased from 50% and 87% in control conditions, to 22% and 62% in the presence of FF (calculated for the 6th and 24th hour of HUVEC/A549 co-cultivation, respectively; Figure 1E). The inhibitory effect of FF on A549-induced HUVEC motility (Figure 1B) and A549 transmigration (Figure 1F) was sensitive to ROS scavenger N-acetyl-L-cysteine (NAC, 1 mM), whereas the PPARα inhibitor (GW6471; 10 µM) had no effect on these parameters (Figure S1A). Co-cultures of HUVEC/A549 cells recapitulate the events involved in A549 diapedesis, sothese observations demonstrate that A549 cells locally deteriorate the endothelial barrier function. FF interferes with A549-induced endothelial cell activation and, consequently, with the diapedesis of A549 cells in a PPARα-independent/ROS-dependent manner.

### 2.2. A549 Cells Impair Endothelial Barrier Function via Intercellular Cx43/EGF/ERK1/2-Dependent Signaling

**Figure 2:**
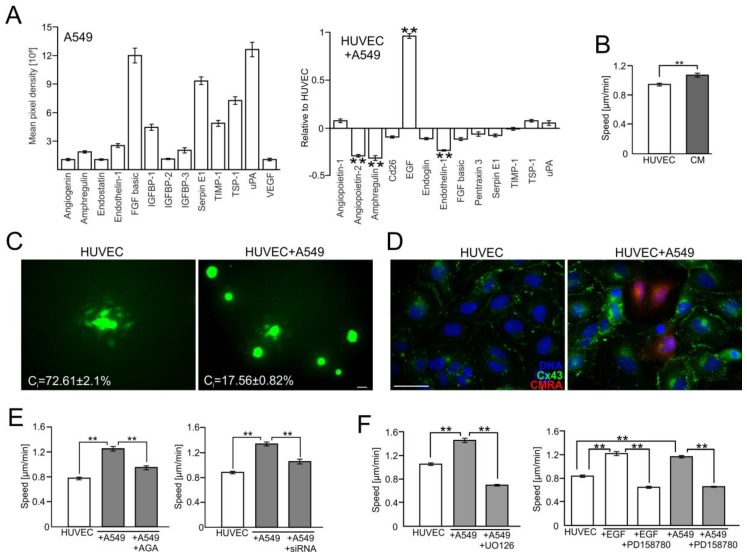
A549 cells impair the endothelial barrier function via the activation of the Cx43/EGF/ERK1/2-dependent intercellular signaling axis. (**A**) A549 cells were seeded onto HUVEC monolayers as in Figure 1 and co-cultured for 24 h. Then, the expression of angioactive proteins was semi-quantitively estimated with an antibody array kit (see Materials and Methods). Plots show the densitometrically estimated dot intensities, illustrating the protein amounts in A549 cells (in a.u.; left) or in A549/HUVEC co-cultures relative to the HUVEC control. (**B**) A549-conditioned medium (3:5) was added to HUVECs and their motility was estimated with time-lapse videomicroscopy for 7 h. (**C**) Calcein-loaded HUVEC (left) or A549 cells (right) were seeded onto HUVEC monolayers and GJIC (coupling ratio-Ci) was estimated by a calcein transfer assay after 1 h. Concomitantly, Cx43 expression in HUVECs and in HUVEC/A549 co-cultures was estimated with immunofluorescence (**D**). (**E**) The effect of AGA (70 μM) and Cx43 silencing by siRNA on HUVEC motility. (**F**) HUVECs were cultured in the presence of EGF or A549/HUVEC co-cultures were established as above and the effects of EGFR- or ERK1/2 inhibitor (PD158780 and UO126, respectively) on HUVEC motility were estimated with time-lapse videomicroscopy. Error bars represent SEM. Scale bar = 40 μm. The statistical significance of the differences was tested with one-way ANOVA followed by post-hoc Tukey’s HSD (**B**,**E**) or non-parametric Dunnett comparison (**A**,**F**); ** *p* < 0.01. All results are representative of at least three independent experiments (*n* ≥ 3). Note the presence of EGF in A549/HUVEC co-cultures and the attenuating effect of chemical Cx43/EGFR and ERK1/2 inhibition on A549-induced HUVEC activation.

To identify the mechanisms underlying the attenuation of the endothelial barrier function by A549 cells, we further focused on the mediators of A549-induced HUVEC activation. Protein array analyses demonstrated the expression of numerous angioactive factors in A549 cells (such as FGF-2, Serpin E1, and uPA), and the up-regulation of EGF in A549/HUVEC co-cultures (Figure 2A). Concomitantly, HUVECs displayed increased motility in A549-conditioned medium (Figure 2B and Figure S1B), which suggests the role of paracrine, EGF-dependent signaling in HUVEC activation by A549 cells. Notably, we also observed a high functionality of gap junctions in HUVEC continua (Figure 2C and Figure S2A). This was accompanied by somewhat limited GJIC between A549 cells and HUVEC, as demonstrated by the relatively low value of a coupling index estimated for HUVEC/A549 co-cultures (C_i_ = 17.6%). A549-induced activation of HUVECs was correlated with an increased abundance of connexin(Cx)43^+^ plaques in HUVEC/A549 co-cultures Cx43 (Figure 2D). Moreover, the inhibition of Cx43-mediated GJIC by 18-α-glicyrrhetinic acid (AGA; 70 μM, cf. Figure S2C in Supplementary data) and Cx43 down-regulation by siRNA (Figure S3) led to the distinct attenuation of HUVEC activation by A549 cells (Figure 2E and Figure S1C,D), in the absence of non-specific effects of control siRNA (Figure S3). Thus, Cx43-mediated communication between A549 cells and HUVECs may up-regulate EGF, which further activates HUVECs in a para/autocrine manner. Actually, ectopic administration of EGF resulted in the activation of HUVECs, whereas chemical inhibition of the EGF receptor (by PD158780, 20 μM) and of ERK1/2 (by UO126, 50 μM) led to the attenuation of this process (Figure 2F; Figures S1E,F and S4). Collectively, these data indicate the involvement of the Cx43/EGF/ERK1/2 axis in A549-induced HUVEC activation.

### 2.3. FF Interferes with Cx43/EGF-Mediated Signaling between HUVECs and A549 Cells

**Figure 3:**
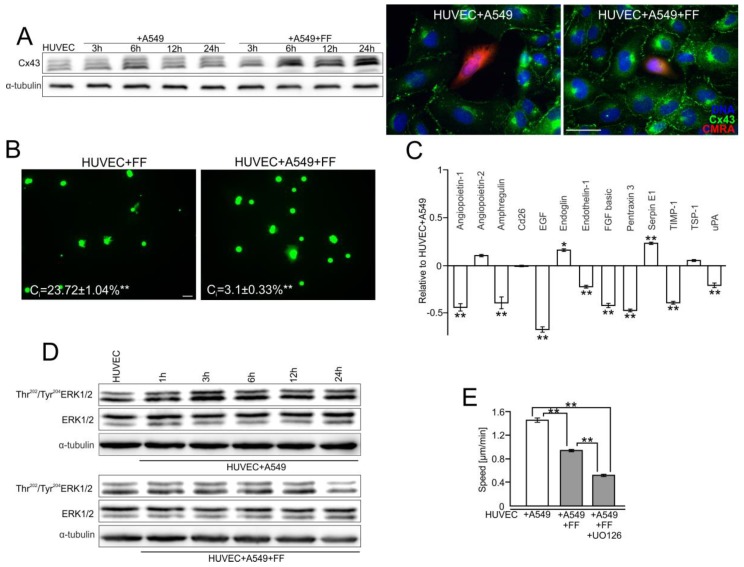
Fenofibrate interferes with the communication networks established between HUVEC and A549 cells. (**A**) HUVEC/A549 co-cultures were established as in Figure 1 and cultivated in the absence or presence of 25 μM FF. Cx43 levels were analyzed at the indicated time points by immunoblotting, quantified by densitometry (Figure S5A,B) and visualized by immunofluorescence after 6 h. (**B**) Calcein-loaded HUVEC (left) or A549 cells (right) were seeded onto HUVEC monolayers and the effect of FF on GJIC (coupling ratio-Ci) was estimated by calcein transfer assay after 1 h. (**C**) Expression of angioactive proteins in FF-treated HUVEC/A549 co-culture was semi-quantitively estimated by the antibody array kit. Plots show the densitometrically estimated dot intensities, illustrating the amounts of a given factor in HUVEC/A549 cells in the presence of 25 μM FF (24 h; relative to HUVEC/A549 control). (**D**) HUVEC/A549 co-cultures were cultivated in the absence or in the presence of 25 μM FF. Tyr^202/204^ERK1/2 levels were analyzed at the indicated time points by immunoblotting and quantified by densitometry (Figure S5C). (**E**) HUVEC/A549 co-cultures were established as above and cultivated in the presence of 25 μM FF. The effect of the ERK1/2 inhibitor (UO126) on HUVEC activation was analyzed with time-lapse videomicroscopy. Error bars represent SEM. Scale bar = 40 μm. The statistical significance of the differences was tested with one-way ANOVA followed by post-hoc Tukey’s HSD (**B**) or non-parametric Dunnett comparison (**A**–**E**); * *p* < 0.05; ** *p* < 0.01. All results are representative of a least three independent experiments (*n* ≥ 3). Note that FF administration inhibits GJIC between cancer and endothelial cells, abolishes the induction of EGF secretion, and inhibits A549-induced motility of HUVECs.

Further analyses were performed to estimate whether FF can inhibit the diapedesis of A549 cells (Figure 1) through an inhibitory effect on the activity of the Cx43/EGF/ERK1/2-dependent axis. The comparison of Cx43 levels in the control and FF-treated HUVEC/A549 co-cultures demonstrated additive effects of A549/FF on endothelial Cx43 expression (Figure 3A and Figure S5A,B). However, they were accompanied by the attenuating effect of FF on GJIC between A549 and endothelial cells, as illustrated by the decreased coupling index (C_i_) in FF-treated A549/HUVEC co-cultures. It decreased from 17.6% in control conditions to 3.1% in the presence of FF (Figure 3B and Figure S2B).

Concomitantly, FF inhibited GJIC within endothelial continua (Figure 3B) and attenuated the expression of angioactive factors, in particular EGF, in A549/HUVEC co-cultures (Figure 3C). It also interfered with A549-induced ERK1/2 activation in HUVECs (Figure 3D and Figure S5C). We also observed the additive inhibitory effect of FF and ERK1/2 inhibitor (UO126) on HUVEC motility (Figure 3E and Figure S1E). These data indicate that FF inhibits A549 diapedesis via the interference with Cx43/EGF/ERK1/2-dependent signaling between HUVECs and A549 cells.

### 2.4. FAK/Rac1/RhoA-Dependent Signaling Participates in the Activation of HUVECs by A549 Cells

**Figure 4:**
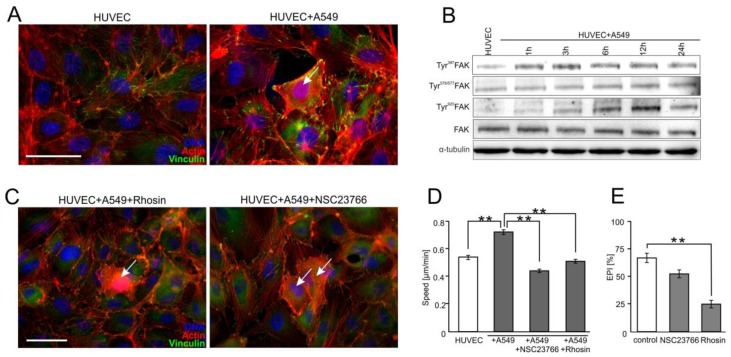
**Focal adhesion kinase** (FAK)/RhoA-dependent signaling participates in the activation of HUVECs by A549 cells. (**A**) A549 cells (marked with arrow) were seeded onto the monolayer of HUVEC as described in Figure 1, cultivated for 6 h, fixed, and stained for F-actin and vinculin. (**B**) A549/HUVEC co-cultures were established as above, and Tyr^397^, Tyr^576/577^, and Tyr^925^ FAK were analyzed at the indicated time points by immunoblotting and quantified by densitometry (Figure S5D). (**C**) Cytoskeletal architecture of HUVEC cultured in co-cultures with A549 cells treated with Rhosin (left) or NSC23766 for 6 h (right). (**D**,**E**) A549/HUVEC co-cultures were treated with Rhosin and NSC23766 followed by the analyses of HUVEC motility (**D**) and A549 diapedesis ((**E**); 6 h). Error bars represent SEM. The statistical significance of the differences was tested with one-way ANOVA followed by post-hoc Tukey’s HSD (**D**,**E**); ** *p* < 0.01. All results are representative of at least three independent experiments (*n* ≥ 3). Scale bar = 40 μm. Note that RhoA/Rac1-dependent disruption of endothelial continuum by A549 cells is accompanied by FAK activation and cytoskeletal rearrangements in proximal HUVECs.

The endothelial barrier function equally depends on the stability of intercellular contacts and the adhesion of endothelial cells to the underlying tissues [8]. Notably, stress fibers’ formation and the maturation of focal contacts were observed in HUVECs co-cultured with A549 cells (Figure 4A). It confirms that Cx43/EGF/ERK1/2-dependent signaling can disturb the equilibrium between the adhesion of endothelial cells to their neighbors and to the extracellular matrix.

To identify the effectors of the Cx43/EGF/ERK1/2-dependent axis during this process, we focused on the activity of focal adhesion kinase (FAK) and small G proteins in HUVECs. Increased phosphorylation of this kinase at Tyr^397^ and Tyr^925^ was observed in HUVECs after A549 seeding. (Figure 4B; Figure S5D in Supplementary data). Analyses of the involvement of RhoA in A549-induced HUVEC activation demonstrated that its chemical inhibition by Rhosin (30 μM) attenuated A549-induced stress fibers formation and the maturation of focal adhesions in HUVECs (Figure 4C). Concomitantly, Rhosin interfered with A549-induced HUVEC motility (Figure 4D and Figure S1G) and inhibited A549 diapedesis (Figure 4E). The inhibition of A549-induced cytoskeletal rearrangements and HUVEC motility was also observed in A549/HUVEC co-cultures treated with Rac1 inhibitor (NSC23766; 50 μM; Figure 4C,D; Figures S1G and S4). However, NSC23766 had minute effects on A549 diapedesis (Figure 4E). Collectively, these observations indicate that the A549-activated Cx43/EGF/ERK1/2-dependent intercellular signaling axis impairs the endothelial barrier function through FAK/RhoA-dependent HUVEC activation. Rac1-dependent signaling plays an auxiliary role in this process.

### 2.5. FF Directly Attenuates HUVEC Susceptibility to the RhoA/Rac1-Dependent Signaling

**Figure 5:**
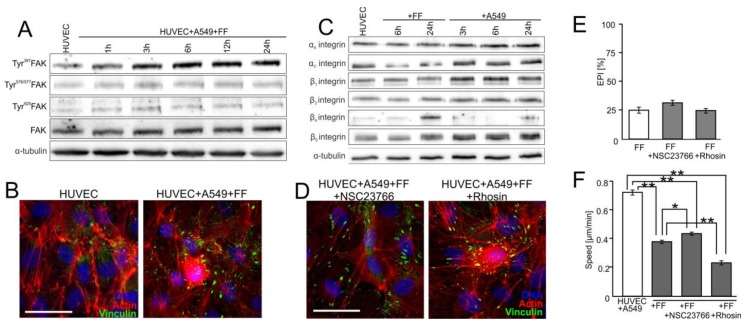
FF directly attenuates HUVEC susceptibility to the activating signals generated by A549 cells. (**A**) HUVEC/A549 co-cultures were established as in Figure 1 and cultivated in the presence of 25 μM FF. Tyr^397^FAK, Tyr^576/577^FAK, and Tyr^925^FAK were analyzed at the indicated time points by immunoblotting and quantified by densitometry (Figure S5E). (**B**) Cytoskeletal architecture in HUVECs (Figure 3A) in A549/HUVEC co-cultures visualized by immunostaining in the absence (left) or presence of 25 μM FF (6 h; right). (**C**) HUVECs were cultivated in the presence of 25 μM FF or A549 cells and the levels of integrins were estimated at the indicated time points by immunoblotting and quantified by densitometry (Figure S5F). (**D**) The effect of NSC23766 (left) or Rhosin (right) on the cytoskeletal architecture of HUVECs cultured in the presence of A549 cells and 25 μM FF (see B for control). (**E**) Effect of NSC23766, Rhosin, and FF on the efficiency of A549 diapedesis (TransEndothelial Penetration; EPI), estimated after 6 h of A549/HUVEC co-culture. Transmigration of at least 200 A549 cells was analyzed for each group. (**F**) Effect of NSC23766 and Rhosin on the motility of proximal HUVECs analyzed in the presence of FF by time-lapse videomicroscopy. Error bars represent SEM. The statistical significance of the differences was tested with one-way ANOVA followed by post-hoc Tukey’s HSD (**E**) or non-parametric Dunnett comparison (**F**); * *p* < 0.05; ** *p* < 0.01 against the control or # *p* < 0.05 against HUVEC/A549 control (see Figure 4B and Figure S5D). All results are representative of at least three independent experiments (*n* ≥ 3). Scale bar = 40 μm. Note that the inhibition of RhoA and Rac1 slightly counteracts FF-induced maturation of focal adhesions while inhibiting A549 diapedesis and HUVEC motility.

FF has previously been shown to augment the endothelial barrier function in the proximity of cancer cells through the direct augmentation of endothelial cells to underlying ECM [9]. Actually, accelerated FAK phosphorylation (Figure 5A and Figure S5E) and augmented maturation of endothelial focal adhesions were observed in FF-treated A549/HUVEC co-cultures (Figure 5B), despite the inhibitory effect of FF on Cx43/EGF/ERK1/2 intercellular signaling between A549 cells and HUVECs (Figure 4).

Concomitantly, the up-regulation of β_3_ and β_4_ integrin was observed in FF-treated HUVECs, whereas A549 cells induced the expression of α_4_, α_v_, β_1_, β_3,_ β_4_, and β_5_ integrin in these cells (Figure 5C and Figure S5F). Furthermore, HUVECs displayed relatively low susceptibility to Rac1/RhoA inhibitors (NSC23766 and Rhosin, respectively) when cultivated in FF-treated co-cultures with A549 cells. This is illustrated by the negligible effects of both agents on FF-augmented HUVEC adhesion (Figure 5D) and FF-inhibited A549 diapedesis (Figure 5E). Only a slightly enhanced maturation of focal contacts was seen in the presence of Rhosin, which correlated with the inhibition of HUVEC motility (Figure 5F and Figure S1G). These observations suggest that FF attenuates the susceptibility of HUVECs to A549-induced activation of Rac1/RhoA-dependent signaling, thus augmenting their barrier function.

### 2.6. FF Selectively Impairs Transendothelial Migration of FibroblastoidA549 Cells

**Figure 6:**
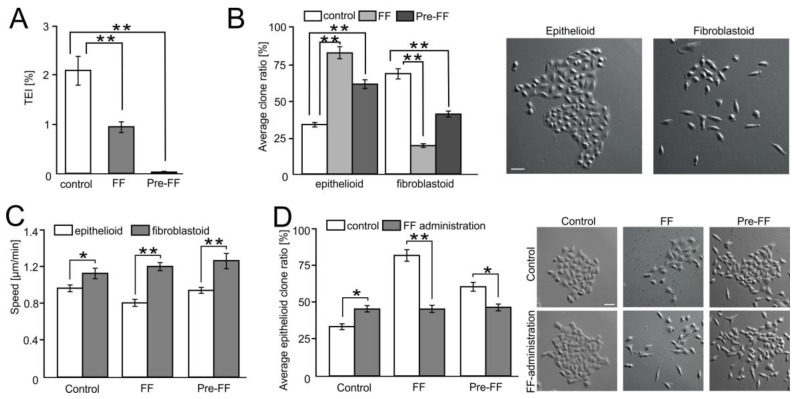
FF selectively impairs the transendothelial migration of fibroblastoid A549 cells. (**A**) A549 cells were seeded on microporous membranes covered by control or FF-pretreated (Pre-FF) HUVEC monolayers, cultivated for 24 h in control conditions or in the presence of 25 μM FF (FF), and the fraction of transmigrated cells was estimated (TransEndothelial penetration Index (TEI)). (**B**) Morphology of A549 microclones derived from the cells that transmigrated through HUVEC-covered microporous membranes in the same conditions as in (**A**). (**C**) Motility of A549 progenies derived from the cells that transmigrated through HUVEC-covered microporous membranes in the same conditions as in (**A**). (**D**) Progenies derived from A549 cells that most readily transmigrated through HUVEC monolayers were treated with FF, and their morphology and the fraction of epithelioid clones was estimated after 72 h. Error bars represent SEM. The statistical significance of the differences was tested with one-way ANOVA followed by post-hoc Tukey’s HSD (**A**,**B**,**D**) or non-parametric Dunnett comparison (**C**); * *p* < 0.05; ** *p* < 0.01. All results are representative of at least three independent experiments (*n* ≥ 3). Scale bar = 40 μm. Note the differential effect of FF on the transmigration of “fibroblastoid” and “epithelioid” A549 cells.

To estimate the biological significance of the augmenting effect of FF on the endothelial barrier function, we further analyzed the selectivity of FF-treated endothelial barriers. For this purpose, we focused on the phenotype of A549 cells that most readily transmigrated through HUVEC-covered microporous membranes. Attenuation of A549 diapedesis, observed in the presence of FF (Figure 1), was accompanied by the decreased efficiency of A549 transmigration through HUVEC-covered microporous membranes (from 2.65% to 1.12% for native HUVECs; and to 0.04% when HUVECs were pre-treated with FF for 6 h; Figure 6A).

A549 cells comprise fibroblastoid and epithelioid sub-populations [34]. Therefore, we further compared the numbers of fibroblastoid and epithelioid cells in the progenies of A549 cells that most readily migrated through HUVEC-covered membranes. A549 cells which transmigrated in the control conditions gave rise to the progeny that was enriched in fibroblastoid cells (Figure 6B). However, a considerably higher fraction of epithelioid cells was found in the progenies of A549 cells transmigrating in the presence of FF. As already mentioned, no differences were observed in the motility of A549 cells propagated in different conditions (Figure 6C), even though a slight dispersion of epithelioid A549 micro-clones was seen in the presence of FF (Figure 6D). Therefore, this effect could hardly be ascribed to the increased motility of epithelioid A549 cells. Collectively, these data indicate that the augmenting effect of FF on the endothelial barrier function selectively inhibits the diapedesis of malignant, fibroblastoid A549 cells.

## 3. Discussion

To form metastases, invasive lung cancer cells must negotiate numerous tissue barriers and withstand defense responses of the organism. Consequently, lung cancer metastases are preferably formed by the progenies of the cells that have gone through numerous cycles of pre-selection. These cells display high adaptability to adverse tissue conditions, relatively high drug-resistance, and invasive potential. This enforces the introduction of aggressive doses of chemotherapeutics that promote a selective expansion of aggressive cancer cell subpopulations and evoke systemic adverse effects. A way out of this vicious circle is given by the metronomic approaches focused on reducing the susceptibility of normal cells to the signals from cancer cells. Importantly, such approaches bypass the drug-resistance of cancer cells without provoking the selection of malignant cancer cell lineages. Here, we described PPARα-independent/ROS-dependent inhibitory effects of 25 μM FF on intercellular signaling between endothelium and lung cancer cells during their diapedesis. This FF concentration remains within the range of maximal serum concentrations of fenofibric acid (an active FF metabolite) in the sera of FF-treated patients [35,36,37]. Due to the crucial role of diapedesis for lung cancer metastasis, our findings open perspectives for the application of this well-tolerated, FDA-approved drug in the treatment of advanced lung cancers.

Extravasation of circulating cancer cells is the final step of their systemic dissemination and the initial point of secondary tumors’ formation [8]. Our data indicate that the diapedesis of A549 cells is initiated by the orchestrated action of GJIC and paracrine signals exchanged between cancer and endothelial cells. The role of cooperative Cx43/EGF-mediated intercellular signaling in this process is illustrated by attenuated endothelial activation in AGA-treated A549/HUVEC co-cultures, the activation of HUVECs upon ectopic EGF administration, and similar HUVEC reactions to PD158780 in the presence of A549 cells and EGF. Accordingly, Cx43/EGF interferes with the endothelial barrier function through the activation of proximal endothelial cells, whereas ErbB2/4, which is also a target for PD158780, does not participate in this axis. Cx43/EGF up-regulation in endothelial cells further enhances intercellular cooperation within the endothelium, thus facilitating endothelial cells’ activation. Additional studies are necessary to elucidate the mechanism underlying A549-induced endothelial Cx43 up-regulation. We have previously shown that endothelial Cx43 up-regulation is accelerated in the proximity of cancer cells that have undergone ectopic Cx43 down-regulation [11]. Therefore, two peaks of Cx43 up-regulation in A549/HUVEC co-cultures may illustrate the consecutive involvement of Cx43-independent and Cx43-dependent mechanisms in this phenomenon. Increased ERK1/2 phosphorylation levels observed during A549 diapedesis and impairment of this process by chemical ERK1/2 inhibition reveals a novel intercellular Cx43/EGF/ERK1/2-dependent signaling axis, which is crucial for lung cancers’ diapedesis. A corresponding Cx43/Akt/ERK1/2/FAK-dependent intercellular pathway has been shown to participate in the diapedesis of prostate cancer cells [9]. Notably, the activation of FAK in A549/HUVEC co-cultures was accompanied by attenuating effects of chemical RhoA/Rac1 inhibition on A549-induced HUVEC activation. Even though the specificity of chemical inhibitors may be limited, these findings indicate that Cx43/EGF/ERK1/2 signaling interferes with the endothelial barrier function via FAK/RhoA/Rac1-dependent deregulation of the balance between endothelial adhesion, contractility, and motility [38].

In our hands, FF interfered with the diapedesis of lung cancer cells via the ROS-dependent inhibition of cooperative Cx43/EGF/ERK1/2-dependent signaling between lung cancer and endothelial cells. This notion is confirmed by the attenuating effect of NAC on the FF-induced inhibition of A549 diapedesis and by reduced levels of Cx43-mediated GJIC, EGF production, and ERK1/2 phosphorylation in FF-treated A549/HUVEC co-cultures. These findings remain in contrast to our previous data [9]. They revealed FF-resistance of a corresponding, Cx43/Akt/ERK1/2/FAK-dependent loop, established between extravasating prostate cancer and endothelial cells. Moreover, different patterns of integrin up-regulation and FAK activation were observed in FF-treated HUVECs and in HUVEC/A549 co-cultures. Thus, FF exerts a direct cytoprotective effect on HUVECs, in addition to its indirect effect on the communication loops between cancer and endothelial cells. Corresponding protective effects of FF on endothelial cells have already been described in vitro and in vivo [12,13,14,15,16,17,18]. In our hands, Rac1/RhoA inhibitors had negligible effects on FF-inhibited HUVEC motility and A549 diapedesis, showing that FF attenuates HUVEC susceptibility to A549-activated FAK/RhoA/Rac1-dependent signaling. These findings confirm that FF augments endothelial adhesion through direct interference with small G protein-dependent cell activation. This effect of FF augments the endothelial barrier function in the proximity of lung cancer cells.

Collectively, our data show the multifaceted augmenting activity of FF on the endothelial barrier function in the proximity of A549 cells. We have identified the intercellular signaling pathway that determines the diapedesis of lung cancer cells. We also revealed the mechanisms underlying the interference of FF with this pathway. Accordingly, connexin(Cx)43-dependent intercellular signaling between A549 cells and HUVECs induces Cx43 expression and the production of EGF in endothelial cells. EGF further activates HUVECs in an ERK1/2-dependent manner and attenuates the endothelial barrier function. Apparently, Cx43 up-regulation within the endothelial continuum facilitates the intracellular propagation of EGF-dependent signals, thus amplifying Cx43/EGF/ERK1/2-dependent signaling throughout proximal endothelium. Other endothelial connexins, including Cx37 and Cx40, may also be involved in this process, even though they are down-regulated in activated endothelia [39]. Consequent activation of FAK/Rac1/RhoA-dependent signaling disturbs the balance between the adhesion, contractility, and motility, which is characteristic of stationary endothelial cells. Thus, it impairs the endothelial barrier function and facilitates cancer cell diapedesis. FF interferes with lung cancer cells’ diapedesis through the activation of ROS-dependent signaling that inhibits the Cx43/EGF/ERK1/2-dependent signaling axis. Concomitantly, it reduces the susceptibility of endothelial cells to FAK/RhoA/Rac1-dependent signaling, thus stabilizing endothelial barriers. Notably, these multifaceted activities of FF lead to the selective transmigration of A549 cells, as illustrated by the reduced fraction of potentially malignant, fibroblastoid cells in the progeny of transmigrating A549 cells. Identification of the mechanisms responsible for this effect remains beyond the scope of this study. However, this effect may additionally impair the metastatic cascade of lung cancer.

Chemotherapeutic strategies focused on the augmentation of tissue barrier functions and/or on the interference with the intercellular communication systems between cancer and endothelial cells are considered as an attractive alternative for traditional chemotherapy. Our data show that FF efficiently interferes with communication networks between lung cancer and endothelial cells, thus potentially inhibiting lung cancer progression. They are consistent with the long list of inhibitory effects of FF on the malignancy of glioma/glioblastoma [29,40], hepatoma [30,41], medulloblastoma [42], melanoma [25,43], and prostate cancer cells [44]. Concomitant augmentation of the endothelial barrier function during the diapedesis can additionally reduce the risk of lung cancer metastases. The application of FF may help to bypass the drawbacks of conventional lung cancer chemotherapy, which are related to systemic adverse effects and to the microevolution of drug-resistant cells. This notion is supported by the low toxicity and high systemic tolerance of fenofibrate, confirmed during decades of its application in the treatment of hyperlipidemia. Studies are now going on to elucidate the consequences of FF for the microevolution of drug-resistant cancer cell sub-populations. They should help to assess whether fenofibrate can be used to establish metronomic treatment regimens of drug-resistant lung tumors.

## 4. Materials and Methods

### 4.1. Cell Cultures

Human umbilical vein endothelial cells (HUVEC; Life Technologies Corporation, Carlsband, CA, USA) were cultured (up to six passages) in endothelial basal medium (EBM; Lonza, Basel, Switzerland) supplemented with 10% fetal bovine serum (FBS) and supplement cocktail (hydrocortisone, recombinant hEGF, bovine brain extract, gentamicin, amphotericin-B; all from Lonza) [9]. Human lung cancer A549 cells (ATCC CCL-185) were cultured in RPMI-1640 medium (Lonza) supplemented with 10% FBS and antibiotics [34]. For co-culture experiments, lung cancer cells were seeded onto HUVEC monolayers (at 70% (cf. 4.3) and 98% confluence (cf. 4.2, 4.4, 4.5,4.6, 4.7)) at the density of 1300 cells/cm^2^. The behavior of HUVEC and A549 cells was analyzed in serum-free EBM medium supplemented with EGF (20 ng/mL; 10605HNAE250, Thermo Fisher Scientific, Waltham, MA, USA), PPARα inhibitor (GW6471; 10 μM; G5045, Sigma, Saint Louis, MO, USA), EGFR inhibitor (PD158780; 20 μM [45,46]; ab141267, Abcam, Cambrige, UK), RhoA inhibitor (Rhosin; 30 μM [46]; 555460, Merck Millipore, Burlington, VT, USA), Rac1-inhibitor (50 μM [47]; NSC23766; 553502, Merck Millipore), ERK1/2 inhibitor (UO126; 50 μM [48]; M 62005, Merck Millipore), 18-α-glicyrrhetinic acid (AGA; 70 μM; G8503, Sigma), fenofibrate (FF; 25 μM F6020, Sigma), and N-acetyl-L-cysteine (NAC; 1 mM; A9165, Sigma). Chemical inhibitors were administrated at the time points indicated in the text and at the concentrations that secure their specific action and the lack of cytotoxic effects (Figure S4). Cell viability was estimated with the Trypan Blue assay (0.4%; Sigma, St. Louis, MO, USA). After trypsinisation (0.25% trypsin, 1 mM EDTA in Ca^2+^/Mg^2+^-free PBS; Sigma, St. Louis, MO, USA), the cells were re-suspended in PBS, mixed with Trypan Blue solution (1:1 *v*/*v*), and analyzed in a haemocytometer. A549-conditioned media (CM) were aspirated from A549 cell cultures after 24 h of incubation, centrifuged (10 min, RT, 1000 rpm), mixed with fresh EBM media (ratio 3:5), and added to HUVEC culture.

### 4.2. Immunofluorescence and Fluorescence Microscopy

For the immunofluorescence analysis, cells were seeded on coverslips as described above. Co-cultures were subsequently fixed with 3.7% formaldehyde at room temperature (RT; 20 min) and permeabilized (0.1% Triton X-100, 5 min). For Cx43 staining, cells were fixed in MetOH/Acetone (7:3) solution for 10 min at −20 °C. Specimens were then blocked with 3% BSA and incubated with a primary antibody (rabbit polyclonal anti-VE-cadherin (1:200; V1514 Sigma), mouse monoclonal anti-vinculin (1:200, V9131 Sigma), rabbit polyclonal anti-TJP1 (ZO-1; 1:200, HPA001636 Sigma), or rabbit polyclonal anti-Cx43 antibody (1:200; C6219; Sigma)) for 1 h. Next, the cells were stained with secondary antibody (AlexaFluor^®^488-conjugated goat anti-mouse IgG (1:300, A11029), AlexaFluor^®^488-conjugated goat anti-rabbit IgG (1:300, A11008), AlexaFluor^®^546-conjugated goat anti-mouse IgG (1:500, A11003); all from Thermo Fisher Scientific), or AlexaFluor^®^488-conjugated phalloidin (1u/slide, A12379 Thermo Fisher Scientific), and counterstained with Hoechst 33358 (0.5 µg/mL, B2883 Sigma). Where indicated, A549 cells were stained with CellTracer Orange CMRA according to the manufacturer’s protocol (10 μM, C34551 Thermo Fisher Scientific). Image acquisition was performed with the Leica DMI6000B microscope (DMI7000 version, Leica Microsystems, Wetzlar, Germany) equipped with the Total Internal Reflection Fluorescence (TIRF) and Nomarski Interference Contrast (DIC) modules.

### 4.3. Cell Motility

The movement of HUVECs in the control condition and in co-cultures with A549 cells was time-lapse recorded using the Leica DMI6000B videomicroscopy system equipped with a temperature chamber (37 °C/5% CO_2_), IMC contrast optics, and a CCD camera. HUVECs were seeded at a density of 500 cells/cm^2^ and cultured for four days to form islets. Then, their movement was recorded for 7 h with 5 min intervals in the absence (control) and the presence of A549 cells (1300/cm^2^) for 7 h. Only HUVECs that had direct contact with A549 cells at the onset of registration were analyzed [11]. The tracks of individual cells were determined from a series of changes in the cell centroid positions. The data were pooled and analyzed to estimate the averaged total length of the cell trajectory (i.e., the “Distance” covered by the cells during the registration time; μm) and the total length of cell displacement (“Displacement”, i.e., the distance from the starting point directly to the cell’s final position; µm), and the average speed of cell movement (Speed; μm/min). Cell trajectories from no less than three independent experiments (number of cells > 50) were obtained for analysis [49]. Instantaneous velocity (V_i_) was calculated at each time point (*t*) according to the formula: V_i_(*t*) = (d_1_ + d_2_)/2Δ*t*, where d_1_ and d_2_ are the distances travelled during two sequential intervals 1 and 2 (between the frame proceeding and the frame following *t*, respectively), and Δ*t* is the time interval between successive frames.

### 4.4. Transendothelial Penetration and Permeability Analyses

HUVECs were seeded on coverslips at 2 × 10^4^ cells/well and grown to confluence for 72 h. Thereafter, A549 cells (1300/cm^2^) stained with CellTracer Orange CMRA (10 μM, Life Technologies) were seeded on HUVEC monolayers and incubated for 6 h and 24 h. Blind microscopic estimation of the percentage of A549 lung cancer cells capable of disrupting the endothelial continuum (transEndothelial Penetration Index-EPI) was performed on F-actin/DNA stained specimens. Transmigration of at least 200 A549 cells was analyzed for each group [50]. For the permeability assay, HUVEC were seeded on Transwell inserts (3 μm pore size, 6.5 mm diameter; Corning) at 1 × 10^5^ cells/insert and non-adherent cells were removed after 6 h. After three days of culture, FF and/or A549 cells were applied to the upper compartments of Transwells in serum-free and phenol red-free medium, followed by the application of FITC-Dextran (MW 42000, 1 mg/mL; after 6 h). Medium samples were taken from the lower compartment after 15, 30, and 60 min and equal volumes of the medium were re-added to the lower chamber. The amount of FITC-Dextran was determined in the samples with an Infinite M200PROmicroplate reader (Tecan Group Ltd., Männedorf, Switzerland), using the excitation wavelength of 492 nm and emission detection at 521 nm [9].

### 4.5. Immunoblots and Array Analyses

Angiogenesis-related protein expression was semi-quantitatively estimated with an antibody array kit (Proteome Profiler^TM^ Human Angiogenesis Array Kit, R&D Systems, Minneapolis, MN, USA), according to the manufacturer’s protocol. Samples were mixed with a cocktail of biotinylated antibodies and then applied onto nitrocellulose membranes to allow their binding to cognate immobilized capture antibodies. Complexes were detected with a streptavidin-HRP system and quantified with the MicroChemii imaging system (Quantity One 1-D Analysis Software, Hercules, CA, USA). The signal was produced at each spot in proportion to the amount of the analyte bound. The results were expressed as the mean pixel density (A549) or fold changes above or below the control indicated in the text. For immunoblot analyses, the cells were dissolved in lysis buffer and cellular proteins (20 μg/lane) were applied to 10% or 15% SDS-polyacrylamide gels, which was followed by transfer to the nitrocellulose membrane. Then, the membranes were exposed to primary antibodies (rabbit polyclonal anti-Cx43 (1:4000; C6219 Sigma), mouse monoclonal anti-α-tubulin (1:2000; T9026 Sigma) IgG, rabbit anti-pERK1/2 (Thr^202^/Tyr^204^; 1:000; 9101 Cell Signaling, Danvers, MA, USA) IgG, rabbit anti-ERK1/2 (1:1000; 9102 Cell Signaling) IgG, rabbit anti-FAK (1:1000; 3285 Cell Signaling) IgG, rabbit anti p-FAK (Tyr^397^, 1:1000; 3283 Cell Signaling) IgG, rabbit anti-p-FAK (Tyr^576/577^, 1:1000; 3281 Cell Signaling) IgG, rabbit anti p-FAK (Tyr^925^, 1:1000; 3284 Cell Signaling) IgG, and rabbit anti-integrin panel (1:1000; 4749 Cell Signaling)), followed by the application of the relevant HRP-labeled secondary antibodies (Life Technologies). HRP activity was detected with Luminata^TM^ Crescendo Western HRP Substrate (Merck Millipore). Total protein analyses were performed on the membranes previously used for the analyses of phosphorylated proteins, subjected to stripping with Western Blot Stripping buffer (Restore^TM^Plus, Thermo Fisher Scientific) [9].

### 4.6. Calcein Transfer Assay

Acceptor HUVEC cells grown in Petri dishes were pre-incubated with FF (25 μM) for 4 h and calcein (Life Technologies, C3099)-loaded donor A549 cells/HUVECs were seeded onto the monolayers of HUVEC cells. After 1 h, calcein transfer from donor to acceptor cells was evaluated using a Leica DM IRE2 inverted fluorescence microscope. Gap junctional intercellular coupling (GJIC) was quantified as the percentage of donor cells, which successfully coupled with the acceptor monolayer (coupling index-C_i_). Dye transfer from at least 200 donor cells per single coverslip was analyzed in threeindependent experiments [51].

### 4.7. Cx43 Silencing by siRNA

A549 cells were seeded at a density of 7 × 10^4^ cells/well into 12-well plates in antibiotic-free RPMI-1640 culture medium supplemented with 10% FBS. After 24 h, the cells were transfected with MISSION^®^GJA1 (EHU105621; 114 pmol; Sigma) or control siRNA (sc-37007, Santa Cruz; 50 nM) using LipofectamineTM2000 (11668019; Invitrogen) according to the manufacturer’s protocol.

### 4.8. Transmigration and Microclone Assay

HUVECs were seeded on Tranwell inserts (8 μm pore size, 6.5 mm diameter; Corning, NY, USA) at a density of 1 × 10^5^ cells/insert and non-adherent cells were removed after 6 h. After threedays of incubation, A549 cells were seeded 1 × 10^4^ onto HUVEC-covered microporous membranes and allowed to transmigrate for the next 24 h in control conditions and in the presence of FF. The TransEndothelial penetration Index (TEI) was estimated as the percentage of seeded cells that penetrated microporous membranes. Where indicated, HUVECs were pre-incubated with 25 μM FF for 6 h before A549 seeding. Progeny of the cells that penetrated the membranes were propagated in standard culture conditions and used for the endpoint experiments. A549 sub-populations cells were seeded at the density of 500 cells/cm^2^ and cultivated for 96 h, and the single-cell-derived microclones were analyzed using the Leica DM IRE2 microscope based on their epithelioid (compact) and fibroblastoid (dispersed) phenotype. The movement of individual cells in the clones was time-lapse recorded for 12 h and the average speed of cell movement (Speed, μm/min) was determined based on the series of changes in the cell centroid position. To compare the sensitivity of established A549 cell sub-populations to FF, the morphology of epithelioid clones was analyzed in control conditions and after 24 h-long incubation in the presence of 25 μM FF using the Leica DMI6000B time-lapse system (Leica Microsystems, Wetzlar, Germany).

### 4.9. Statistical Analysis

All data were expressed as mean ± SEM from at least three independent experiments (*n* = 3). The statistical significance was tested with one-way ANOVA followed by post-hoc Dunnett’s or Tukey’s comparison for variables with a non-normal (tested with Levene’s comparison) and normal distribution, respectively. Statistical significance was shown at ^#,^* *p* < 0.05; ^##,^** *p* < 0.01.

## 5. Conclusions

Collectively, our data show that lung cancer cells locally activate adjacent endothelium and impair the endothelial barrier function via cooperative gap junction-dependent and paracrine signaling. Fenofibrate impairs communication between lung cancer and endothelial cells and directly augments endothelial cell adhesion, thus selectively impairing the diapedesis of malignant lung cancer cells. Thus, FF can be used for metronomic treatment of lung cancer in elderly patients.

## Supplementary Materials

The following are available online at http://www.mdpi.com/2072-6694/10/10/363/s1, Figure S1: Single cell analyses of HUVEC motility, Figure S2: Gap junctional intercellular coupling in A549/HUVEC populations, Figure S3: Effect of Cx43siRNA and control siRNA on Cx43 expression in A549 cells, Figure S4: Effect of chemical inhibitors on the viability and instantaneous speeds of HUVECs, Figure S5: Densitometric evaluation and statistical analysis of immunoblot data; Video S1: Diapedesis control; Video S2: Diapedesis FF.

## References

[1] Siegel R.L., Miller K.D., Jemal A. (2016). Cancer statistics, 2016. CA Cancer J. Clin..

[2] Torre L.A., Siegel R.L., Jemal A. (2016). Lung Cancer Statistics. Adv. Exp. Med. Biol..

[3] Savagner P. (2010). The epithelial-mesenchymal transition (EMT) phenomenon. Ann. Oncol..

[4] Wang X., Ferreira A.M., Shao Q., Laird D.W., Sandig M. (2005). Beta3 integrins facilitate matrix interactions during transendothelial migration of PC3 prostate tumor cells. Prostate.

[5] Woodward J. (2008). Crossing the endothelium: E-selectin regulates tumor cell migration under flow conditions. Cell Adhes. Migr..

[6] Strell C., Niggemann B., Voss M.J., Powe D.G., Zanker K.S., Entschladen F. (2012). Norepinephrine promotes the beta1-integrin-mediated adhesion of MDA-MB-231 cells to vascular endothelium by the induction of a GROalpha release. Mol. Cancer Res..

[7] Van Zijl F., Krupitza G., Mikulits W. (2011). Initial steps of metastasis: Cell invasion and endothelial transmigration. Mutat. Res..

[8] Reymond N., d’Agua B.B., Ridley A.J. (2013). Crossing the endothelial barrier during metastasis. Nat. Rev. Cancer.

[9] Piwowarczyk K., Wybieralska E., Baran J., Borowczyk J., Rybak P., Kosinska M., Wlodarczyk A.J., Michalik M., Siedlar M., Madeja Z. (2014). Fenofibrate enhances barrier function of endothelial continuum within the metastatic niche of prostate cancer cells. Expert Opin. Ther. Targets.

[10] Czyz J., Piwowarczyk K., Paw M., Luty M., Wrobel T., Catapano J., Madeja Z., Ryszawy D. (2017). Connexin-dependent intercellular stress signaling in tissue homeostasis and tumor development. Acta Biochim. Pol..

[11] Piwowarczyk K., Paw M., Ryszawy D., Rutkowska-Zapala M., Madeja Z., Siedlar M., Czyz J. (2017). Connexin43high prostate cancer cells induce endothelial connexin43 up-regulation through the activation of intercellular ERK1/2-dependent signaling axis. Eur. J. Cell Biol..

[12] Goetze S., Eilers F., Bungenstock A., Kintscher U., Stawowy P., Blaschke F., Graf K., Law R.E., Fleck E., Grafe M. (2002). PPAR activators inhibit endothelial cell migration by targeting Akt. Biochem. Biophys. Res. Commun..

[13] Varet J., Vincent L., Mirshahi P., Pille J.V., Legrand E., Opolon P., Mishal Z., Soria J., Li H., Soria C. (2003). Fenofibrate inhibits angiogenesis in vitro and in vivo. Cell. Mol. Life Sci..

[14] Meissner M., Stein M., Urbich C., Reisinger K., Suske G., Staels B., Kaufmann R., Gille J. (2004). PPARalpha activators inhibit vascular endothelial growth factor receptor-2 expression by repressing Sp1-dependent DNA binding and transactivation. Circ. Res..

[15] Panigrahy D., Kaipainen A., Huang S., Butterfield C.E., Barnes C.M., Fannon M., Laforme A.M., Chaponis D.M., Folkman J., Kieran M.W. (2008). PPARalpha agonist fenofibrate suppresses tumor growth through direct and indirect angiogenesis inhibition. Proc. Natl. Acad. Sci. USA.

[16] Cao Z., Shang B., Zhang G., Miele L., Sarkar F.H., Wang Z., Zhou Q. (2013). Tumor cell-mediated neovascularization and lymphangiogenesis contrive tumor progression and cancer metastasis. Biochim. Biophys. Acta.

[17] Hida K., Ohga N., Akiyama K., Maishi N., Hida Y. (2013). Heterogeneity of tumor endothelial cells. Cancer Sci..

[18] Robison N.J., Campigotto F., Chi S.N., Manley P.E., Turner C.D., Zimmerman M.A., Chordas C.A., Werger A.M., Allen J.C., Goldman S. (2014). A phase II trial of a multi-agent oral antiangiogenic (metronomic) regimen in children with recurrent or progressive cancer. Pediatr. Blood Cancer.

[19] Grabacka M., Reiss K. (2008). Anticancer Properties of PPARα-Effects on Cellular Metabolism and Inflammation. PPAR Res..

[20] Grabacka M., Pierzchalska M., Reiss K. (2013). Peroxisome proliferator activated receptor α ligands as anticancer drugs targeting mitochondrial metabolism. Curr. Pharm. Biotechnol..

[21] Robinson J.G. (2008). LDL reduction: How low should we go and is it safe?. Curr. Cardiol. Rep..

[22] Adeghate E., Adem A., Hasan M.Y., Tekes K., Kalasz H. (2011). Medicinal Chemistry and Actions of Dual and Pan PPAR Modulators. Open. Med. Chem. J..

[23] Balakumar P., Rohilla A., Mahadevan N. (2011). Pleiotropic actions of fenofibrate on the heart. Pharmacol. Res..

[24] McKeage K., Keating G.M. (2011). Fenofibrate: A review of its use in dyslipidaemia. Drugs.

[25] Grabacka M., Plonka P.M., Urbanska K., Reiss K. (2006). Peroxisome proliferator-activated receptor alpha activation decreases metastatic potential of melanoma cells in vitro via down-regulation of Akt. Clin. Cancer Res..

[26] Thuillier P., Anchiraico G.J., Nickel K.P., Maldve R.E., Gimenez-Conti I., Muga S.J., Liu K.L., Fischer S.M., Belury M.A. (2000). Activators of peroxisome proliferator-activated receptor-alpha partially inhibit mouse skin tumor promotion. Mol. Carcinog..

[27] Saidi S.A., Holland C.M., Charnock-Jones D.S., Smith S.K. (2006). In vitro and in vivo effects of the PPAR-α agonists fenofibrate and retinoic acid in endometrial cancer. Mol. Cancer.

[28] Panigrahy D., Kaipainen A., Kieran M.W., Huang S. (2008). PPARs: A Double-Edged Sword in Cancer Therapy?. PPAR Res..

[29] Drukala J., Urbanska K., Wilk A., Grabacka M., Wybieralska E., Del Valle L., Madeja Z., Reiss K. (2010). ROS accumulation and IGF-IR inhibition contribute to fenofibrate/PPARα -mediated inhibition of Glioma cell notility in vitro. Mol. Cancer.

[30] Jiao H.L., Zhao B.L. (2002). Cytotoxic effect of peroxisome proliferator fenofibrate on human HepG2 hepatoma cell line and relevant mechanisms. Toxicol. Appl. Pharmacol..

[31] Scatena R., Bottoni P., Giardina B. (2008). Mitochondria, PPARs, and Cancer: Is Receptor-Independent Action of PPAR Agonists a Key?. PPAR Res..

[32] Czyz J., Szpak K., Madeja Z. (2012). The role of connexins in prostate cancer promotion and progression. Nat. Rev. Urol..

[33] Piwowarczyk K. (2011). Personal observation.

[34] Bechyne I., Szpak K., Madeja Z., Czyz J. (2011). Functional heterogeneity of non-small lung adenocarcinoma cell sub-populations. Cell Biol. Int..

[35] Doser K., Guserle R., Nitsche V., Arnold G. (1996). Comparative steady state study with 2 fenofibrate 250 mg slow release capsules. An example of bioequivalence assessment with a highly variable drug. Int. J. Clin. Pharmacol. Ther..

[36] Kajosaari L.I., Backman J.T., Neuvonen M., Laitila J., Neuvonen P.J. (2004). Lack of effect of bezafibrate and fenofibrate on the pharmacokinetics and pharmacodynamics of repaglinide. Br. J. Clin. Pharmacol..

[37] Uetake D., Ohno I., Ichida K., Yamaguchi Y., Saikawa H., Endou H., Hosoya T. (2010). Effect of fenofibrate on uric acid metabolism and urate transporter 1. Intern. Med..

[38] Huveneers S., Danen E.H. (2009). Adhesion signaling–crosstalk between integrins, Src and Rho. J. Cell Sci..

[39] Kwak B.R., Mulhaupt F., Veillard N., Gros D.B., Mach F. (2002). Altered pattern of vascular connexin expression in atherosclerotic plaques. Arterioscler. Thromb. Vasc. Biol..

[40] Giordano A., Macaluso M. (2012). Fenofibrate triggers apoptosis of glioblastoma cells in vitro: New insights for therapy. Cell. Cycle.

[41] Yamasaki D., Kawabe N., Nakamura H., Tachibana K., Ishimoto K., Tanaka T., Aburatani H., Sakai J., Hamakubo T., Kodama T. (2011). Fenofibrate suppresses growth of the human hepatocellular carcinoma cell via PPARalpha-independent mechanisms. Eur. J. Cell Biol..

[42] Urbanska K., Pannizzo P., Grabacka M., Croul S., Del Valle L., Khalili K., Reiss K. (2008). Activation of PPARalpha inhibits IGF-I-mediated growth and survival responses in medulloblastoma cell lines. Int. J. Cancer.

[43] Grabacka M., Placha W., Plonka P.M., Pajak S., Urbanska K., Laidler P., Slominski A. (2004). Inhibition of melanoma metastases by fenofibrate. Arch. Dermatol. Res..

[44] Wybieralska E., Szpak K., Gorecki A., Bonarek P., Miekus K., Drukala J., Majka M., Reiss K., Madeja Z., Czyz J. (2011). Fenofibrate attenuates contact-stimulated cell motility and gap junctional coupling in DU-145 human prostate cancer cell populations. Oncol. Rep..

[45] Purdom S., Chen Q.M. (2005). Epidermal growth factor receptor-dependent and -independent pathways in hydrogen peroxide-induced mitogen-activated protein kinase activation in cardiomyocytes and heart fibroblasts. J. Pharmacol. Exp. Ther..

[46] Zimolag E., Borowczyk-Michalowska J., Kedracka-Krok S., Skupien-Rabian B., Karnas E., Lasota S., Sroka J., Drukala J., Madeja Z. (2017). Electric field as a potential directional cue in homing of bone marrow-derived mesenchymal stem cells to cutaneous wounds. Biochim. Biophys. Acta.

[47] Gao Y., Dickerson J.B., Guo F., Zheng J., Zheng Y. (2004). Rational design and characterization of a Rac GTPase-specific small molecule inhibitor. Proc. Natl. Acad. Sci. USA.

[48] Horne M.M., Guadagno T.M. (2003). A requirement for MAP kinase in the assembly and maintenance of the mitotic spindle. J. Cell Biol..

[49] Piwowarczyk K., Sarna M., Ryszawy D., Czyz J. (2017). Invasive Cx43high sub-line of human prostate DU145 cells displays increased nanomechanical deformability. Acta Biochim. Pol..

[50] Ryszawy D., Sarna M., Rak M., Szpak K., Kedracka-Krok S., Michalik M., Siedlar M., Zuba-Surma E., Burda K., Korohoda W. (2014). Functional links between Snail-1 and Cx43 account for the recruitment of Cx43-positive cells into the invasive front of prostate cancer. Carcinogenesis.

[51] Szpak K., Wybieralska E., Niedzialkowska E., Rak M., Bechyne I., Michalik M., Madeja Z., Czyz J. (2011). DU-145 prostate carcinoma cells that selectively transmigrate narrow obstacles express elevated levels of CX43. Cell. Mol. Biol. Lett..

