# Supplementary material for: Fenofibrate Interferes with the Diapedesis of Lung Adenocarcinoma Cells through the Interference with Cx43/EGF-Dependent Intercellular Signaling
Source: Cancers (Basel). 2018 Sep 28;10(10):363. doi: 10.3390/cancers10100363 (PMC6210471; doi:10.3390/cancers10100363)
Supplement: Supplementary file 1 [file cancers-10-00363-s001.zip › cancers-357385-suppl.-proof/cancers-357385-suppl.-proof-.doc]

Supplementary Materials: Fenofibrate Interferes with the Diapedesis of Lung Adenocarcinoma Cells Through the Interference with Cx43/EGF-Dependent Intercellular Signaling

Katarzyna Piwowarczyk, Edyta Kwiecień, Justyna Sośniak, Eliza Zimoląg, Emiliana Guzik, Jolanta Sroka, Zbigniew Madeja and Jarosław Czyż

**Figure S1.** Single cell analyses of HUVEC motility. A549 cells were seeded onto the monolayer of HUVECs at the density of 1300 cells/cm^2^. HUVEC motility was visualized by a time-lapse videomicroscopy in the conditions ascertaining their basal motility (70% confluence). Dot-plots show movement parameters of proximal cells at the single cell level (registered for 7 h; *n* > 50). The data were pooled and analyzed to estimate the average length of cell trajectory; (Distance; μm), the average length of cell displacement i.e., the distance from the starting point directly to the cell’s final position (Displacement; μm). Results are representative of three independent experiments. Where indicated, A549 cells were subjected to Cx43 down-regulation (by siRNA), or HUVECs were pretreated with EGF (20 ng/mL), FF (25 μM), GW6471 (10 μM), NAC (1 mM), HUVEC/A549-conditioned medium, AGA (70 μM), UO126 (50 μM), PD158780 (20 μM), NSC23766 (50 μM), or Rhosin (30 μM), and cultivated in the presence/absence of A549 cells.

**Figure S2.** Gap junctional intercellular coupling in A549/HUVEC populations. Calcein/DiI-loaded donor HUVEC or A549 cells were seeded onto the monolayers of acceptor A549 cells or HUVECs (see PhC images) in the absence (**A**) and in the presence of 25 μM FF (**B**), or in the presence of 70 μM AGA. The transfer of calcein (green) from DiI-stained donor (red) to DiI-negative acceptor cells was visualized with fluorescence microscopy after 1 h (right column). All results are representative of at least 3 independent experiments. Scale bar = 40 μm.

**Figure S3.** Effect of Cx43siRNA and control siRNA on Cx43 expression in A549 cells and on endothelial Cx43 up-regulation. A549 cells were seeded at a density of 7 × 10^4^ cells/well in antibiotic-free RPMI-1640 culture medium supplemented with 10% FBS. After 24 h, the cells were transfected with MISSION^®^GJA1 or control siRNA (sc-37007, Santa Cruz, CA, USA) using LipofectamineTM2000 (11668019; Invitrogen) and subjected to immunoblot analyses at the indicated time points. Note the lack of the effect of control siRNA on the ability of A549 cells to induce endothelial Cx43 up-regulation.

**Figure S4.** Effect of chemical inhibitors on the susceptibility of HUVECs to the signals from A549 cells and on their viability. (**A**) Instantaneous speeds of single HUVEC in contact with A549 cells registered in the presence of indicated chemical inhibitors (quantified based on changes in cell centroid position registered for 7 h by time-lapse videomicroscopy). * show the positions of analyzed cells at the starting and ending point of analysis. Scale bar = 40 μm. (**B**) Effect of chemical inhibitors on the viability of HUVECs estimated by trypan blue assay. At least 200 cells per single coverslip were analyzed in 3 independent experiments.

**Figure S5.** Densitometric evaluation and statistical analysis of immunoblot data. (**A**) Effect of A549 lung cancer cells and FF on endothelial Cx43 expression levels in HUVECs (see Figure 3A). (**B**) Synergy of A549/FF effects on endothelial Cx43 expression levels in HUVECs (see Figure 3A). (**C**) The effect of 25 μM FF on ERK1/2 phosphorylation levels in HUVEC/A549 co-cultures (see Figure 3D). (**D**) The effect of A549 cells on endothelial FAK phosphorylation (see Figure 4B). (**E**) Effect of 25 μM FF on endothelial FAK phosphorylation in HUVEC/A549 co-cultures (see Figure 5A). (**F**) Effect of A549 cells and 25 μM FF on the expression of integrins in HUVECs (see Figure 5C). Band intensities (ROD) were calculated in relation to the corresponding control (α-tubulin) using the ImageJ gel analysis tool. The statistical significance of the differences vs. HUVEC (100%, *) and HUVEC/A549 (E,C, #) was tested with one-way ANOVA followed by post-hoc Tukey’s HSD (**B**) or non-parametric Dunnett comparison (**A**,**C**–**F**). ^#,^* *p* < 0.05, ^##,^** *p* < 0.01. All results are representative of at least 3 independent experiments.
